# Differential glycemic effects of DWP16001 in diabetic dogs according to baseline glycemic status: a multicenter randomized controlled trial

**DOI:** 10.1186/s12917-025-04962-y

**Published:** 2025-08-12

**Authors:** Ju-Hyun An, Byung-Gee Ko, Hyun Namkung, Hye-Gyu Lee, Hyun-Woo Lim, Wan Huh, Joon Seok Park, Jae-Bong Moon, Hwa-Young Youn, Min-Ok Ryu

**Affiliations:** 1 Department of Veterinary Emergency and Critical Care Medicine, Institute of Veterinary Science, College of Veterinary Medicine, Kangwon National University, Chuncheon, Republic of Korea; 2 Laboratory of Internal Medicine, Department of Veterinary Clinical Science, College of Veterinary Science, Seoul National University, Seoul, 08826 Republic of Korea; 3 Global Business Center, Daewong Pharmaceutical Co., Ltd., Seoul, Republic of Korea; 4 Daewoong Pharmaceutical, Life Science Institute, Yongin, Republic of Korea; 5 Daewoong pet, Seoul, Republic of Korea

**Keywords:** Dogs, DWP16001, Diabetes mellitus, Sodium-glucose cotransporter-2 inhibitor

## Abstract

**Background:**

Sodium-glucose cotransporter 2 inhibitors are widely used in human medicine for their insulin-independent glucose-lowering effects. However, their clinical efficacy and safety for managing diabetes mellitus in dogs have not been established. This study aimed to evaluate the efficacy and safety of DWP16001, a selective sodium-glucose cotransporter 2 inhibitor, as an adjunct to insulin therapy for client-owned dogs with diabetes mellitus.

**Methods:**

This multicenter, randomized, double-blind, placebo-controlled clinical trial involved 61 dogs receiving stable insulin therapy. They were randomly assigned to receive DWP16001 (0.025 mg/kg PO q24h) or placebo for 8 weeks. The changes in the serum fructosamine (weeks 0, 1, 4, and 8) and HbA1c (weeks 0, 4 and 8) concentrations and daily insulin dose were assessed. Safety evaluations included adverse event monitoring, physical examination, body weight measurement, blood gas and ketone analyses, urinalysis, and hematologic and biochemical profiling. Hypoglycemia was detected via blood glucose curve.

**Results:**

DWP16001 significantly reduced fructosamine and HbA1c concentrations in the dogs, especially in those with poor baseline glycemic control (fructosamine ≥ 500 µmol/L, HbA1c ≥ 6%). A trend toward reduced insulin requirements was observed in the treatment group without significant weight loss or clinically relevant changes in systolic blood pressure or signs of volume depletion. Asymptomatic hypoglycemia was detected in four dogs receiving DWP16001, but it resolved following insulin dose adjustment. No episodes of diabetic ketoacidosis were recorded, and laboratory parameters remained stable throughout the study.

**Conclusions:**

DWP16001 is a safe and effective adjunct to insulin therapy for diabetic dogs, especially those with suboptimal glycemic control. It demonstrated insulin-sparing effects and favorable metabolic safety, necessitating further evaluation in long-term clinical studies.

## Introduction

 Sodium-glucose cotransporter 2 (SGLT-2) inhibitors are a novel class of oral antidiabetic agents that lower blood glucose by inhibiting renal glucose reabsorption in the proximal tubules. Unlike treatments that act by enhancing insulin sensitivity or supplying exogenous insulin, SGLT-2 inhibitors lower blood glucose via an insulin-independent mechanism, by promoting urinary glucose excretion through inhibition of renal glucose reabsorption in the proximal tubules [1]. In addition to their primary role in glycemic control, SGLT-2 inhibitors have demonstrated several effects in human patients, including reductions of body weight, blood pressure, and cardiovascular disease risk [2, 3].

These agents are commonly referred to as gliflozins, based on their chemical class. Enavogliflozin—also known as DWP16001 in veterinary applications—is one such selective SGLT-2 inhibitor. Originally developed for human use, enavogliflozin has shown favorable pharmacokinetic and safety profiles, and DWP16001 represents its investigational use in canine patients with diabetes mellitus.

Their use has expanded rapidly in recent years, and they are now considered a cornerstone in the management of type 2 diabetes mellitus. They have also been shown to be effective for treating heart failure and chronic kidney disease [4, 5].

Diabetes mellitus is a common endocrine disorder in dogs, and it is predominantly insulin-dependent (similar to type 1 diabetes in humans). Management typically involves exogenous insulin administration combined with dietary regulation [6]. However, achieving optimal glycemic control can be challenging due to factors such as owner compliance, variability in insulin pharmacokinetics—including absorption and duration of action—and individual metabolic differences. Moreover, while insulin therapy is necessary for glycemic control, it does not directly resolve several associated metabolic abnormalities, such as glycosuria, dyslipidemia, increased protein catabolism, and oxidative stress, which may persist despite treatment. In addition, long-term insulin use may lead to complications such as hypoglycemia [7]. These limitations highlight the need for adjunctive or alternative therapeutic strategies in canine diabetes management.

Despite the clinical success of SGLT-2 inhibitors in human medicine, their use in veterinary patients remains limited. Enavogliflozin (DWP16001), a highly selective SGLT-2 inhibitor with over 2000-fold selectivity for SGLT-2 over SGLT-1, has demonstrated favorable pharmacokinetic and safety profiles in preclinical human studies [8, 9]. Preliminary studies and anecdotal case reports [10, 11] have suggested that these agents may offer similar glycemic and metabolic benefits in canine diabetic patients by promoting insulin-independent urinary glucose excretion. In addition, recent studies have shown that SGLT-2 inhibitors, such as velagliflozin [12] and bexagliflozin [13], may be beneficial in diabetic cats, which more frequently exhibit insulin resistance. While SGLT-2 inhibitors have also been explored as adjunctive therapies in human patients with type 1 diabetes mellitus [14–16], their use in this population remains cautious due to the potential risk of euglycemic diabetic ketoacidosis. However, comprehensive studies assessing the pharmacokinetics, efficacy, and safety profile of SGLT-2 inhibitors in dogs are currently lacking.”

Considering the unmet need for adjunctive therapies in the management of canine diabetes mellitus and the promising pharmacological profile of SGLT-2 inhibitors, this study investigated the potential of DWP16001, a selective SGLT-2 inhibitor. We aimed to evaluate the safety and efficacy of DWP16001 in improving glycemic control in insulin-treated diabetic dogs with suboptimal glycemic regulation, defined in this study as dogs with elevated baseline fructosamine concentrations (≥ 500 µmol/L) and/or HbA1c values > 6%.

## Materials and methods

### Study design and animals

This prospective, multicenter, double-blind, randomized, placebo-controlled clinical trial involved client-owned dogs of various breeds (e.g., Miniature Poodle, Maltese, Miniature Pinscher, Miniature Schnauzer, and mixed breeds) diagnosed with naturally occurring diabetes mellitus. Both investigators and dog owners were blinded to treatment allocation. DWP16001 and placebo tablets were identical in appearance and packaging and were dispensed using subject codes assigned through centralized randomization. Dogs were randomly assigned in a 1:1 ratio to receive either DWP16001 or placebo using a randomization schedule prepared by an independent third party who was not involved in the clinical procedures or data analysis. Allocation was concealed, and treatment assignments were not disclosed to investigators or owners until the completion of the study to ensure blinding.

Dogs were eligible for enrollment if they met al.l of the following inclusion criteria: a stable insulin dosage within ± 10% variation over the preceding month, a blood β-hydroxybutyrate (BHB) concentration of ≤ 0.5 mmol/L [10], and provision of written informed consent by the owner. Blood ketone concentrations were measured using point-of-care (POC) ketone meters available at each participating institution, all of which detect BHB and are validated for clinical use. No dietary restrictions were imposed, and dogs remained on their usual diets, which included both prescription diabetic diets and general commercial maintenance diets. Owners were instructed to maintain consistent feeding times and avoid any major dietary changes throughout the study period.

The exclusion criteria included a body condition score (BCS) of < 3 on a 9-point scale; administration of diuretics (e.g., furosemide, spironolactone) or medications with significant diuretic effects; diagnosis of renal failure classified as International Renal Interest Society (IRIS) stage ≥ 3; oliguria or anuria; diagnosis of endocrine disorders (such as hypoadrenocorticism, hyperadrenocorticism, or hypothyroidism) that were newly diagnosed, poorly controlled, or required treatment adjustments within the past 6 months; ongoing corticosteroid therapy; history of diabetic ketoacidosis or hypoglycemia requiring emergency treatment within the past 3 months; intact females or dogs that were pregnant, in estrus, or lactating; and use of prescription or over-the-counter medications within 14 days before enrollment that, in the opinion of the investigator, could affect study outcomes or animal safety.

Eligible dogs were randomized to receive DWP16001 (0.025 mg/kg PO q24h) or placebo for 8 weeks, with the dosage based on a previous pilot study [10]. The placebo consisted of an oral formulation identical in appearance to the DWP16001 tablets but contained only inert ingredients and no active compound. Both the study drug and placebo were administered orally once daily with meals.

Owners were instructed to observe for clinical signs of hypoglycemia (e.g., lethargy, weakness, seizures) or poor glycemic control (e.g., polyuria, polydipsia, weight loss), and report any concerns immediately to the attending clinicians. Routine home glucose monitoring was not required during the study. All glycemic assessments, including blood glucose curves and laboratory evaluations, were performed at scheduled hospital visits. Insulin dosage was maintained throughout the study unless clinically indicated. If marked hypo- or hyperglycemia occurred, insulin adjustments were made at the discretion of the attending veterinarian, but standardized titration protocols were not applied in order to reflect real-world clinical practice.

### Efficacy and safety assessment

Efficacy was assessed by serial measurements of serum fructosamine concentrations at baseline (week 0) and weeks 1, 4, and 8. Fructosamine concentration was selected as the primary efficacy endpoint. Changes in fructosamine concentrations from the baseline in the treatment and placebo groups were compared. Serum fructosamine concentrations were measured using the Solo analyzer (Eurolyser, Salzburg, Austria), following the manufacturer’s instructions. The exploratory efficacy variables included glycated hemoglobin (HbA1c) concentrations and insulin dosage. The HbA1c concentration was measured at weeks 4 and 8. HbA1c concentrations were measured using a point-of-care veterinary-specific analyzer (Osang Healthcare, Anyang, Republic of Korea). The changes in insulin dosage for the participants in both groups were documented at each visit (weeks 1, 4, and 8) based on daily dosing records and blood glucose curves obtained using point-of-care (POC) glucometers, which varied by institution. These data were compared to evaluate the potential insulin-sparing effects of DWP16001.

Safety assessments included monitoring for adverse events (especially hypoglycemia, defined as blood glucose < 60 mg/dL; urinary tract infection, assessed via urinalysis and confirmed by urine culture when indicated; and diabetic ketoacidosis, defined as the presence of hyperglycemia, ketonemia, and metabolic acidosis), as well as physical examination findings, body weight, and vital signs at each visit (weeks 0, 1, 4, and 8). Clinical pathology evaluations included complete blood count (CBC), serum biochemistry, blood gas analysis, and measurement of blood β-hydroxybutyrate (BHB) levels. CBC was analyzed using the XN-1000 V analyzer (Sysmex, Kobe, Japan), and serum biochemistry including SDMA was measured using the Cobas C702 analyzer (Roche Diagnostics, Basel, Switzerland). Blood gas analysis was performed using point-of-care (POC) analyzers available at each participating institution. Blood BHB concentrations were assessed using POC handheld ketone meters, which varied by institution but were validated for clinical use and designed to detect circulating BHB, the predominant ketone body. To evaluate the renal and glycosuric effects of DWP16001 treatment, urine protein-to-creatinine ratio (UPC) was measured at the central laboratory using commercial services (IDEXX Laboratories, Westbrook, ME, USA), and urine specific gravity (USG) was determined using refractometers at each local site. Urine glucose and ketone levels were assessed semi-quantitatively using dipstick tests, and results were reported as negative, trace, or 1 + to 4+. Values reported as below the lower limit of detection (LOD) of the assay were substituted with one-half of the LOD (LOD/2) for statistical analysis, in accordance with commonly accepted practices in biomedical research.

A blood glucose curve was performed for at least 8 h at 2-hour intervals to detect hypoglycemic events and assess overall glycemic trends. Owners were instructed to observe their dogs closely for any clinical signs of adverse effects and report any abnormalities immediately. In the event of a severe adverse reaction, the investigator was responsible for initiating appropriate clinical care and determining whether the dog should be withdrawn from the study.

All other necessary medical care was permitted during the study, except the use of prohibited concurrent medications.

### Clinical scoring

A customized scoring system (Table 1) was used to monitor general clinical signs and potential adverse effects throughout the study. This system included six key parameters commonly altered in diabetic dogs: appetite, activity, fecal consistency, vomiting, water intake, and urination. Although not validated for use in diabetic dogs, it was designed to facilitate consistent tracking of clinical trends during treatment.

**Table 1:** Clinical scoring criteria for monitoring the general condition of dogs

Parameter	Score	Description
Appetite	0	No change (normal food intake)
	1	Increased (more than usual)
	−1	Decreased (≤ 75% of usual intake)
	−2	None (refuses even preferred food)
Activity	0	No change (normal activity)
	1	Increased (more active than usual)
	−1	Decreased (lethargic or less responsive)
	−2	None (no voluntary movement)
Fecal Consistency	1	Firm (dry and well-formed)
	0	Normal (moist, retains shape)
	−1	Soft (partially formed, moist)
	−2	Diarrhea (liquid or unformed stool)
Vomiting	0	None
	−1	Once per week
	−2	2–3 times per week
	−3	More than 3 times per week
Water Intake	0	No change (normal consumption)
	1	Increased (drinks more than usual)
	−1	Decreased (drinks less than usual)
Urination	0	No change (normal frequency)
	1	Increased (more frequent than usual)
	−1	Decreased (less frequent than usual)

### Statistical analysis

Data were analyzed using GraphPad Prism 6.01 (GraphPad Software, San Diego, CA). Data distribution was assessed using the Shapiro–Wilk test to determine normality. Continuous variables with a normal distribution were analyzed using two-way analysis of variance (ANOVA) to evaluate treatment and time effects, followed by Bonferroni correction for post-hoc comparisons. Ordinal variables, such as clinical scores, and non-normally distributed data were analyzed using non-parametric tests (e.g., Mann–Whitney U test for between-group comparisons and Wilcoxon signed-rank test for within-group comparisons). A p-value < 0.05 was considered statistically significant.

## Results

### Patient information

Among the 64 dogs initially recruited for this study, three were lost to follow-up and excluded from the study analysis. Of the remaining 61, 30 were allocated to the DWP16001 group, while 31 were allocated to the placebo group (Table 2). The median age was 10 years for the DWP16001 group and 9 years for the placebo group. Both groups had a median BCS of 5. All dogs had been treated with insulin for at least 3 months prior to enrollment, although the exact duration was not consistently documented. The mean insulin dose at baseline was 2.00 ± 1.26 U/kg/day for the DWP16001 group and 1.99 ± 1.27 U/kg/day for the placebo group, with no statistically significant difference between groups. Regarding insulin type, NPH insulin was the most commonly used, with 15 dogs in the DWP16001 group and 13 in the placebo group receiving it. Lente porcine insulin (Caninsulin^®^, MSD Animal Health) was used in 10 dogs in the DWP16001 group and 9 dogs in the placebo group. A minority of dogs were treated with other insulin types, including glargine (5 in the DWP16001 group and 7 in the placebo group) and detemir (2 in the placebo group). Detailed information on insulin types is summarized in Table 2.

**Table 2:** Baseline characteristics of dogs in the DWP16001 and placebo groups

	DWP16001 group (*n* = 30)	Placebo group (*n* = 31)
Breeds (n)	Bichon Frise (1), Dachshund (1), Maltese (10), Miniature Pinsher (1), Mix (2), Miniature Poodle (14), Miniature Schnauzer (1)	Chihuahua (1), Siberian Husky (1), Maltese (11), Miniature Pinsher (1), Mix (2), Pomeranian (3), Miniature Poodle (6), Pug (1), Miniature Schnauzer (1), Shih-Tzu (1), Yorkshire Terrier (3)
Sex (n)	CM (20), SF (9), M (1)	CM (21), SF (9), M (1)
Age (median, range)	10 (4–14)	9 (5–14)
body weight (median, range)	4.94 (3.1–10)	5.2 (2.88-23)
Body condition score (median, range)	5 (3–7)	5 (4–8)
Comorbidity (n)	Hyperadrenocorticism (3), MMVD (3), hypothyroidism (3), MUE (1), IVDD (1)	Hyperadrenocorticism (7), MMVD (3), hypothyroidism (1)
Insulin type (n)	lente porcine insulin (10), NPH (15), Glargine (5)	lente porcine insulin (9), Detemir (2), NPH (13), Glargine (7)
Insulin dose (U/kg/day, mean ± SD)	2.00 ± 1.26	1.99 ± 1.27
Fructosamine (µmol/L)	567.19 ± 94.64	458.31 ± 61.37 ****
HbA1c (%)	5.98 ± 0.9	5.95 ± 0.81

Data are presented as number (n), median (range), or mean ± standard deviation (SD), as appropriate. No statistically significant differences were observed between treatment groups for age, body weight, body condition score, insulin dose or HbA1c (*p* > 0.05 for all comparisons). Fructosamine concentrations showed a statistically significant difference between the two groups (****p < 0.0001). *CM* castrated male, *MMVD* myxomatous mitral valve disease, *MUE* meningoencephalitis of unknown etiology, *SF* spayed female

### Effects of DWP16001 on body weight, body condition score, blood pressure, insulin dose and hypoglycemic event

**Fig. 1:**
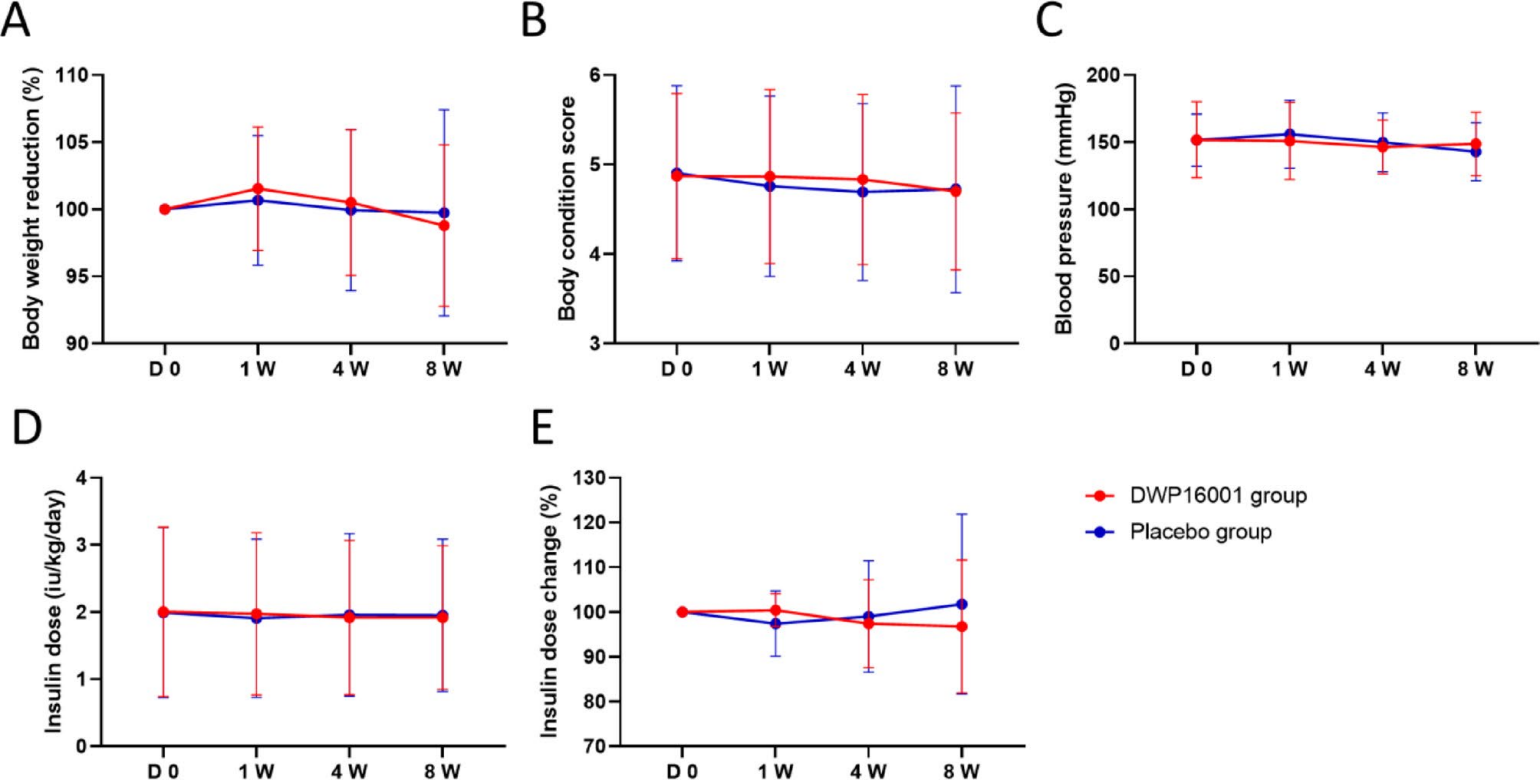
DWP16001 effects on body weight, body condition, blood pressure, and insulin dose in diabetic dogs. Longitudinal changes in **A** body weight reduction (%), **B** body condition score, **C** systolic blood pressure (mmHg), **D** insulin dose (U/kg/day), and **E** insulin dose (%) over 8 weeks in dogs treated with DWP16001 (red) or placebo (blue). Data are expressed as mean ± SD. No significant differences were observed between groups at any time point. DWP16001 treatment showed trends of stabilization in body weight and insulin dose change relative to placebo. *n* = 30 DWP16001 group, *n* = 31 Placebo group

During the 8 weeks of treatment, both DWP16001 and placebo groups maintained relatively stable clinical and metabolic parameters, with modest variations (Fig. 1). Body weight (% of baseline) remained largely consistent for both groups. The mean body weight slightly decreased from 100% at baseline to 98.78 ± 6.01 at week 8 for the DWP16001 group (*p* = 0.9129) and from 100% to 99.74 ± 7.68 for the placebo group (*p* > 0.9999). No statistically significant weight loss was observed in either group (*p* = 0.9957) (Fig. 1A). BCS was assessed using a 9-point scale, with 1 indicating emaciation, 9 indicating severe obesity, and 4–5 considered ideal. The BCS was consistent throughout the study. The DWP16001 group maintained a stable BCS (from 4.87 ± 0.94 at baseline to 4.7 ± 0.88 at 8 weeks, *p* = 0.7929), while the placebo group showed minimal fluctuation (from 4.90 ± 0.98 to 4.73 ± 1.15, *p* = 0.7577) (Fig. 1B). As a result of measuring systolic blood pressure (mmHg), The DWP16001 group showed a slight decrease from baseline (151.8 ± 28.2 mmHg) to 8 weeks (148.67 ± 23.64 mmHg) (*p* > 0.9999). The placebo group demonstrated greater variability with a decrease from 151.5 ± 19.5 to 142.9 ± 21.6 mmHg by week 8 (*p* > 0.9999), although the difference was not statistically significant (Fig. 1C).

To assess changes in insulin dosage and glucose profiles following the administration of DWP16001, blood glucose curves were obtained at screening, week 1, week 4, and week 8 for all 61 participating dogs. All 61 enrolled dogs were receiving insulin twice daily (q12h, BID) at baseline. Changes in insulin requirements were evaluated based on the total daily insulin dose (U/kg/day), as all dogs followed the same dosing frequency. Eighteen dogs had changes in insulin dosage over the 8 weeks from the baseline. Of these, 5 (1 in the DWP16001 group and 4 in the placebo group) required an increase in insulin dose, while 13 (6 in the DWP16001 group and 7 in the placebo group) required a decrease. The mean daily insulin dosage (U/kg/day) slightly reduced for both groups. In the DWP16001 group, the dose decreased from 2.00 ± 1.26 at baseline to 1.92 ± 1.07 at week 8, while the placebo group showed a similar decline from 1.99 ± 1.27 to 1.95 ± 1.13. However, these changes were not statistically significant over time, and no significant differences were observed between the DWP16001 and placebo groups at any individual time point (Fig. 1D).

The percentage change in insulin dosage (%) also suggested a trend toward dose reduction for the DWP16001 group, with the insulin dosage decreasing to 96.77 ± 14.84% of the baseline value; however, this was also not statistically significant (Fig. 1E). Additionally, analysis of the glucose curves revealed that 5 dogs (4 in the DWP16001 group and 1 in the placebo group) had blood glucose concentrations below 80 mg/dL. While values below this threshold do not necessarily indicate true hypoglycemia, they may raise clinical concern. No clinical signs consistent with hypoglycemia were observed in any of these dogs. As a precautionary measure, insulin dosages were reduced in all affected cases. Although the number of dogs with low glucose values was described, no statistical comparison between groups was performed.

### Clinical signs monitoring

**Fig. 2:**
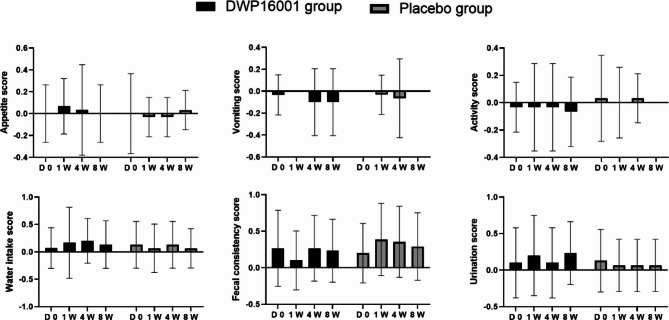
Changes in subjective symptom scores over time in the DWP16001 and placebo groups. Mean changes from baseline in **A** appetite score, **B** vomiting score, **C** activity score, **D** water intake score, **E** fecal consistency score, and **F** urination score were evaluated at baseline (D0), 1 week (1 W), 4 weeks (4 W), and 8 weeks (8 W) following administration. Black bars represent the DWP16001 treatment group, and grey bars represent the placebo group. Data are presented as mean ± standard deviation (SD)

Across the 8 weeks of study, no significant differences in the clinical signs were observed between the DWP16001 and placebo groups. All parameters remained relatively stable, with only minor fluctuations. At week 8, although not statistically significant, the DWP16001 group showed a trend toward increased water intake and urination scores compared to the placebo group, which may reflect the expected osmotic effects of SGLT2 inhibition. Overall, both the treatment and placebo groups had no clinically meaningful adverse effects, and DWP16001 administration appeared to be well tolerated based on the monitored clinical indicators (Fig. 2).

### Hematological, serum biochemical and venous blood gas analysis parameter

No significant differences were observed in all hematological, serum biochemical, and venous blood gas analysis parameters between the DWP16001 and placebo groups throughout the study. The detailed results are presented in Table 3.

**Table 3:** Hematologic, biochemical, and venous blood gas parameters

Parameter	Reference range	Group	D 0	1 W	4 W	8 W
Complete blood count						
RBC (×10⁶/µL)	5.65–8.87	DWP16001	6.91 ± 0.99	6.67 ± 1.13	6.81 ± 1.01	7.11 ± 1.04
		Placebo	7.10 ± 1.04	7.14 ± 0.94	7.25 ± 0.90	7.23 ± 0.92
HCT (%)	37.3–61.7	DWP16001	48.66 ± 5.64	48.00 ± 6.56	48.75 ± 5.99	50.63 ± 6.10
		Placebo	50.00 ± 6.69	50.45 ± 4.89	50.74 ± 5.29	51.01 ± 4.05
PLT (×10³/µL)	148–484	DWP16001	399.43 ± 180.34	378.80 ± 156.19	390.73 ± 141.93	364.20 ± 155.41
		Placebo	385.81 ± 153.21	381.39 ± 163.58	393.87 ± 150.24	366.27 ± 151.98
WBC (×10³/µL)	5.05–16.76	DWP16001	11.51 ± 4.19	44.93 ± 195.32	9.80 ± 3.05	9.41 ± 2.88
		Placebo	10.57 ± 3.49	13.03 ± 3.45	10.35 ± 3.40	9.81 ± 3.46
Serum chemistry						
TP (g/dL)	5.2–8.2	DWP16001	6.24 ± 0.45	6.33 ± 0.49	6.35 ± 0.52	6.30 ± 0.51
		Placebo	6.07 ± 0.36	6.14 ± 0.42	6.01 ± 0.48	6.11 ± 0.40
ALB (g/dL)	2.3–4.4	DWP16001	3.75 ± 0.36	3.81 ± 0.35	3.86 ± 0.31	3.87 ± 0.32
		Placebo	3.67 ± 0.31	3.73 ± 0.30	3.68 ± 0.29	3.68 ± 0.32
ALT (U/L)	12–121	DWP16001	105.27 ± 70.18	89.80 ± 72.61	71.30 ± 61.55	71.77 ± 53.76
		Placebo	91.23 ± 66.60	84.39 ± 58.22	96.85 ± 53.75	88.77 ± 53.49
AST (U/L)	5–55	DWP16001	32.92 ± 20.37	27.90 ± 15.66	25.40 ± 17.60	27.23 ± 13.96
		Placebo	35.87 ± 25.36	35.35 ± 18.90	32.23 ± 15.04	30.85 ± 13.87
ALP (U/L)	11–212	DWP16001	588.77 ± 556.27	518.40 ± 530.24	417.20 ± 546.65	442.00 ± 582.56
		Placebo	447.03 ± 491.32	452.68 ± 739.63	445.50 ± 726.00	380.93 ± 441.81
GGT (U/L)	0–14	DWP16001	14.52 ± 26.40	14.52 ± 20.07	13.21 ± 19.76	14.77 ± 22.19
		Placebo	15.18 ± 23.08	20.77 ± 20.07	20.25 ± 23.47	15.75 ± 18.00
T-bil (mg/dL)	0–0.5	DWP16001	0.06 ± 0.05	0.07 ± 0.08	0.07 ± 0.06	0.08 ± 0.06
		Placebo	0.06 ± 0.06	0.06 ± 0.05	0.05 ± 0.04	0.04 ± 0.04
Amylase (U/L)	500–1500	DWP16001	543.93 ± 376.27	482.80 ± 248.97	539.27 ± 533.51	477.10 ± 225.71
		Placebo	430.03 ± 150.44	498.26 ± 284.36	461.77 ± 266.47	422.84 ± 141.43
Lipase (U/L)	0–250	DWP16001	173.33 ± 283.86	136.80 ± 139.79	172.67 ± 290.76	146.77 ± 178.92
		Placebo	102.39 ± 226.98	150.77 ± 182.94	117.31 ± 92.71	91.52 ± 85.27
Tchol (mg/dL)	110–320	DWP16001	351.37 ± 144.36	216.30 ± 151.66	202.33 ± 92.46	212.38 ± 166.66
		Placebo	348.06 ± 108.99	358.00 ± 147.19	361.45 ± 211.76	319.13 ± 141.84
TG (mg/dL)	10–150	DWP16001	545.90 ± 192.45	496.20 ± 186.08	525.27 ± 205.91	490.05 ± 166.66
		Placebo	358.94 ± 108.99	339.45 ± 147.19	351.61 ± 211.76	355.41 ± 141.84
BUN (mg/dL)	6–31	DWP16001	21.50 ± 12.14	27.43 ± 17.61	26.00 ± 15.34	24.15 ± 13.59
		Placebo	20.23 ± 9.66	21.71 ± 9.58	25.60 ± 10.84	22.61 ± 12.01
CREA (mg/dL)	0.5–1.6	DWP16001	0.83 ± 0.43	0.81 ± 0.23	0.78 ± 0.25	0.75 ± 0.25
		Placebo	0.62 ± 0.15	0.63 ± 0.20	0.64 ± 0.16	0.67 ± 0.17
IP (mg/dL)	2.5–6.8	DWP16001	4.09 ± 1.11	4.21 ± 1.45	4.20 ± 1.07	3.89 ± 1.06
		Placebo	3.79 ± 1.02	3.89 ± 0.96	3.26 ± 0.80	3.21 ± 0.91
SDMA (µg/dL)		DWP16001	8.60 ± 2.95	10.73 ± 4.49	10.87 ± 3.53	9.26 ± 3.23
		Placebo	8.08 ± 2.67	8.26 ± 2.44	8.41 ± 2.38	8.06 ± 4.11
Na (mmol/L)	139–154	DWP16001	142.57 ± 5.53	144.80 ± 3.33	144.80 ± 4.47	144.93 ± 4.67
		Placebo	142.35 ± 3.46	143.19 ± 3.55	142.06 ± 4.71	144.00 ± 3.83
K (mmol/L)	3.6–5.8	DWP16001	5.15 ± 0.62	5.02 ± 0.59	4.94 ± 0.52	4.87 ± 0.54
		Placebo	4.67 ± 0.99	5.17 ± 0.60	4.95 ± 0.62	4.85 ± 0.59
Cl (mmol/L)	103–120	DWP16001	103.80 ± 5.92	106.57 ± 4.17	102.77 ± 17.83	106.27 ± 4.13
		Placebo	101.96 ± 17.51	101.30 ± 17.17	103.87 ± 4.22	104.87 ± 4.87
Ketone (mmol/L)	< 0.4	DWP16001	0.16 ± 0.11	0.28 ± 0.75	0.27 ± 0.66	0.40 ± 1.00
		Placebo	0.22 ± 0.23	0.16 ± 0.18	0.20 ± 0.20	0.25 ± 0.71
Venous blood gas analysis						
pH	7.31–7.46	DWP16001	7.39 ± 0.04	7.40 ± 0.06	7.40 ± 0.04	7.43 ± 0.13
		Placebo	7.39 ± 0.05	7.39 ± 0.06	7.40 ± 0.06	7.41 ± 0.05
HCO₃ (mmol/L)		DWP16001	23.74 ± 3.16	23.01 ± 4.59	23.50 ± 3.35	23.94 ± 2.69
		Placebo	22.09 ± 5.05	23.34 ± 5.16	23.60 ± 3.65	22.32 ± 4.44
AG (mmol/L)	10–20	DWP16001	14.17 ± 4.77	14.25 ± 3.57	13.83 ± 4.05	13.25 ± 4.07
		Placebo	13.93 ± 5.45	14.05 ± 4.55	14.71 ± 5.18	14.49 ± 5.20

Measurements were taken at baseline (D0), and at weeks 1 (1 W), 4 (4 W), and 8 (8 W) following treatment. No statistically significant differences were observed between the DWP16001 and placebo groups at any time point

Data are presented as mean ± SD. *ALB* albumin, *ALP* alkaline phosphatase, *ALT* alanine aminotransferase, *Amylase* amylase, *AST* aspartate aminotransferase, *BUN* blood urea nitrogen, *Cl* chloride, *CREA* creatinine, *GGT* gamma-glutamyl transferase, *HCT* hematocrit, *IP* inorganic phosphate, *K* potassium, *Lipase* lipase, *Na* sodium, *PLT* platelet count, *RBC* red blood cell count, *SDMA* symmetric dimethylarginine, *T-bil* total bilirubin, *Tchol* total cholesterol, *TG* triglyceride, *TP* total protein, *WBC* white blood cell count

### Effects of DWP16001 on urinary parameters

For the DWP16001-treated group, the mean UPC decreased from 0.96 ± 2.12 at baseline to 0.38 ± 0.58 at week 1 and remained low through weeks 4 (0.40 ± 0.50) and 8 (0.46 ± 0.66). However, the change was not significant. In contrast, the UPC values demonstrated non-significant fluctuations over time, with an increase noted at week 4 (1.42 ± 0.93) before decreasing again at week 8 (0.67 ± 0.83). The USG values remained consistent across all time points for both groups. For the DWP16001 group, USG was maintained at 1.04 ± 0.01 throughout the 8 weeks. Similar stability was observed for the placebo group. Urine glucose concentrations were elevated for both groups across all time points. For the DWP16001 group, urine glucose scores assessed by dipstick increased from 3.10 ± 1.16 (D0) to a peak of 3.67 ± 0.66 at week 4. These values represent semi-quantitative dipstick scores on a 0 to 4 scale (negative = 0, trace = 0.5, 1 + = 1, etc.) rather than absolute concentrations. A similar trend was observed for the placebo group but with slightly lower average values at all time points. No statistically significant differences were observed between the groups. Urine ketone positivity was sporadically observed across both groups and time points, with no consistent pattern or marked increase related to DWP16001 treatment. Most dogs remained negative or trace-positive (1+), and no signs of diabetic ketoacidosis were detected throughout the study (Table 4).

**Table 4:** Changes in UPC, USG, and urine glucose and ketone concentrations over time in the DWP16001 and placebo groups

Parameter	Group	D 0	1 W	4 W	8 W
UPC (Mean ± SD)	DWP16001	0.96 ± 2.12	0.38 ± 0.58	0.40 ± 0.50	0.46 ± 0.66
	Placebo	0.88 ± 1.40	0.70 ± 0.93	0.96 ± 1.42	0.67 ± 0.83
USG (Mean ± SD)	DWP16001	1.04 ± 0.012	1.04 ± 0.011	1.04 ± 0.010	1.04 ± 0.008
	Placebo	1.04 ± 0.013	1.04 ± 0.012	1.04 ± 0.013	1.04 ± 0.010
Urine glucose (Mean ± SD)	DWP16001	3.10 ± 1.16	3.50 ± 0.94	3.67 ± 0.66	3.57 ± 0.77
	Placebo	3.03 ± 1.25	3.06 ± 1.06	3.00 ± 1.21	3.23 ± 0.88
Urine ketone (n)	DWP16001	1+ (2), 2+ (1), 3+ (1)	1+ (1)	1+ (2)	1+ (3)
	Placebo	1+ (3), 4+ (1)	2+ (1)	1+ (1)	1+ (1)

Measurements were taken at baseline (D0), and at weeks 1 (1 W), 4 (4 W), and 8 (8 W) following treatment. No statistically significant differences were observed between the DWP16001 and placebo groups in UPC, USG, or urine glucose at any time point. Statistical analysis was not performed for urine ketone levels due to their categorical nature and small sample counts

Data are presented as mean ± SD

### Effects of DWP16001 on fructosamine and HbA1c concentrations

At baseline, fructosamine concentrations were significantly higher in the DWP16001 group than in the placebo group (*p* < 0.0001), whereas HbA1c concentrations did not differ significantly between groups (*p* = 0.8549). For the entire DWP16001 group (*n* = 30), the fructosamine concentrations were significantly reduced at weeks 1, 4, and 8 relative to the baseline, indicating an early and sustained glucose-lowering effect of DWP16001. To better assess treatment response, dogs were stratified based on baseline fructosamine concentrations (≥ 500 µmol/L vs. < 500 µmol/L), which are commonly used to define suboptimal versus adequate glycemic control. There were no significant differences in baseline fructosamine concentrations between the DWP16001 and placebo groups within each subgroup. Dogs with baseline fructosamine concentrations of ≥ 500 µmol/L (*n* = 21) showed a marked reduction at all time points post-treatment (*p* < 0.0001). In contrast, dogs with baseline fructosamine concentrations of < 500 µmol/L (*n* = 9) showed no significant change during the 8 weeks, suggesting that the glucose-lowering effect was more pronounced in dogs with inadequate glycemic control, as reflected by elevated baseline fructosamine concentrations. For the placebo group, no significant changes in fructosamine concentrations were observed in the entire population (*n* = 31) or in baseline concentration-based subgroups (≥ 500 or < 500 µmol/L). In addition, there were no statistically significant differences in fructosamine concentrations between the DWP16001 and placebo groups at any individual time point (weeks 1, 4, or 8), either in the total population or in the subgroups stratified by baseline fructosamine concentrations.

**Fig. 3:**
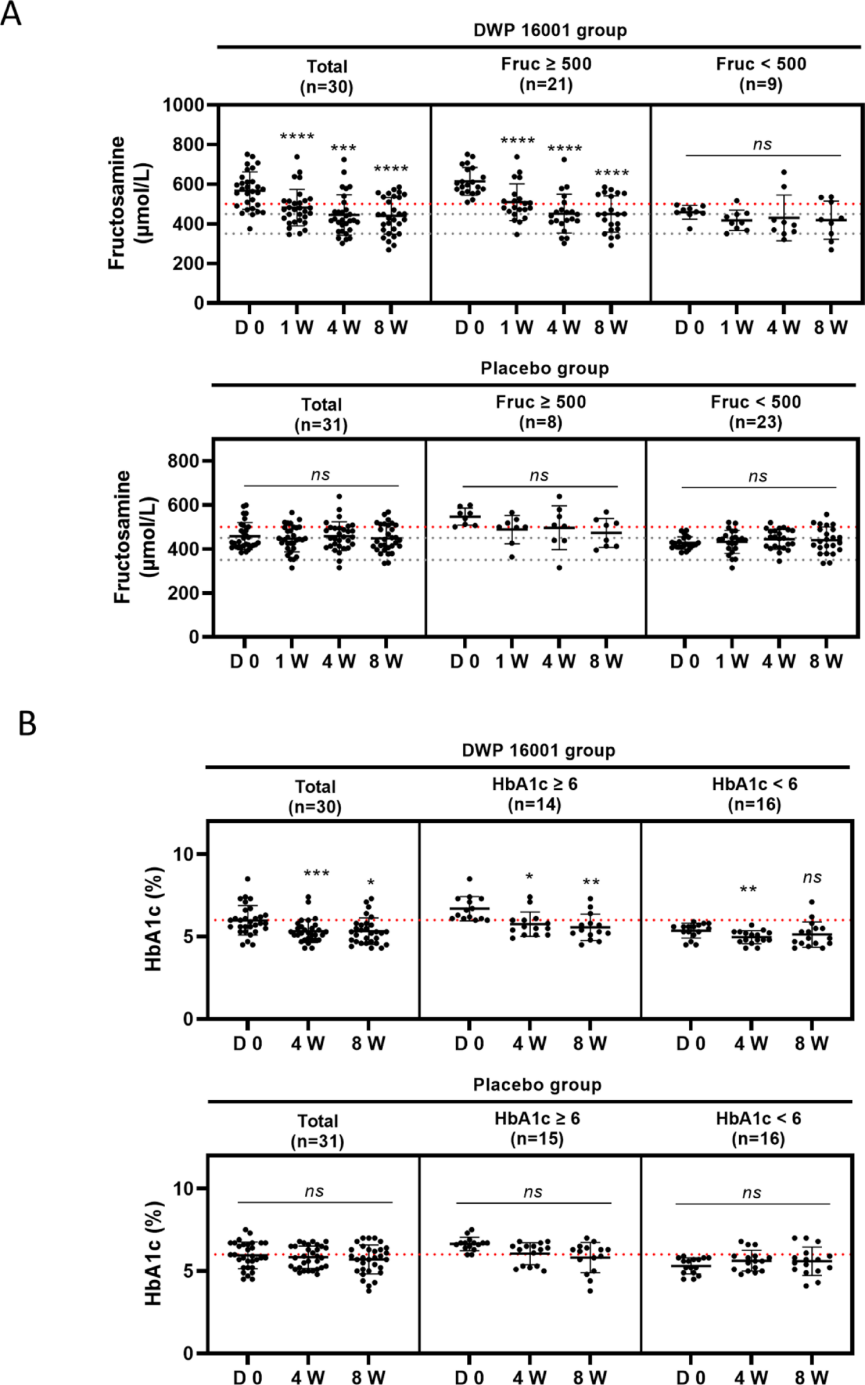
DWP16001 effects on glycemic control markers (fructosamine and HbA1c) in diabetic dogs over 8 weeks. **A** Fructosamine concentrations (µmol/L) were measured at baseline (D 0), 1 week (1 W), 4 weeks (4 W), and 8 weeks (8 W) in the DWP16001 group (*n* = 30) and the placebo group (*n* = 31). Subgroups were divided based on baseline fructosamine concentrations (≥ 500 vs. < 500 µmol/L). **B** HbA1c (%) concentrations were assessed at baseline, 4 weeks, and 8 weeks. Subgroups were divided based on baseline HbA1c concentrations (≥ 6 vs. < 6%). Data are presented as mean ± SD. Statistical significance was evaluated versus baseline (D 0): *p < 0.05, **p < 0.01, ***p < 0.001, ****p < 0.0001; *ns* not significant

For the entire group (*n* = 30), HbA1c concentrations significantly decreased at week 4 (*p* < 0.001) and remained low at week 8 (*p* < 0.05). To evaluate treatment response according to glycemic status, dogs were stratified by baseline HbA1c concentrations using 6% as the cutoff value. Subgroup analysis based on baseline HbA1c concentrations revealed a significant reduction at weeks 4 (*p* < 0.05) and 8 (*p* < 0.01) in dogs with concentrations of ≥ 6% (*n* = 14). Dogs with HbA1c concentrations of < 6% (*n* = 16) showed a modest but statistically significant reduction at week 4 (*p* < 0.01), which was not sustained at week 8 (ns). The placebo group did not show significant changes in HbA1c concentrations during the study in either the entire population or subgroups (all ns) (Fig. 3). In addition, there were no statistically significant differences in HbA1c concentrations between the DWP16001 and placebo groups at any individual time point (weeks 4, or 8), either in the total population or in the subgroups stratified by baseline HbA1c concentrations.

## Discussion

This study evaluated the efficacy and safety of DWP16001, a sodium-glucose cotransporter 2 (SGLT2) inhibitor, in diabetic dogs receiving insulin therapy. The primary aim was to determine whether the addition of DWP16001 could improve glycemic control, reduce insulin dosage, and maintain physiological and clinical stability in treated dogs.

Fructosamine and glycated hemoglobin (HbA1c) were evaluated as markers of glycemic control to provide complementary insights into short- to intermediate-term glucose regulation. While fructosamine reflects glycemic status over the past 2–3 weeks, HbA1c represents average glucose levels over a longer duration, typically 2–3 months.

A key finding of this study was the significant reduction in fructosamine and HbA1c concentrations in the DWP16001-treated group, suggesting an early and sustained glucose-lowering effect. The effect was more pronounced in dogs with elevated baseline fructosamine (≥ 500 µmol/L) and HbA1c concentrations (≥ 6%), suggesting that DWP16001 may be particularly beneficial in dogs with presumed higher average glucose levels and suboptimal glycemic control. These cutoff values were selected based on previously reported reference intervals and clinical thresholds for suboptimal glycemic control in dogs. Fructosamine concentrations above 500 µmol/L are generally considered indicative of persistent hyperglycemia over the previous 2–3 weeks [17], while HbA1c concentrations exceeding 6% have been associated with inadequate long-term glycemic control in canine patients [18]. Therefore, these criteria were used to identify individuals most likely to benefit from adjunctive glucose-lowering therapy. These results are consistent with prior studies in human medicine [5], where SGLT2 inhibitors have demonstrated enhanced efficacy in patients with higher baseline glucose concentrations. The use of SGLT2 inhibitors in the treatment of diabetes reduces plasma glucose concentrations in proportion to the current blood glucose concentration and the glomerular filtration of glucose. Although SGLT2 inhibitors are known to promote glucosuria and thereby reduce blood glucose levels, no statistically significant difference in urine glucose concentrations was observed between the treatment and placebo groups in this study. Nevertheless, the greater glucose-lowering effect observed in dogs with higher baseline fructosamine and HbA1c levels may still reflect the glucose-dependent mechanism of action of SGLT2 inhibitors, highlighting the potential for a tailored approach to their clinical use.

The changes in daily insulin dose did not reach statistical significance, but a trend toward insulin dose reduction was observed in the DWP16001 group. This observation aligns with trends reported in previous studies [10]. During an earlier investigation, oral administration of DWP16001 at a dose of 0.025 mg/kg once daily for 8 weeks to diabetic dogs was associated with a non-significant tendency toward weight reduction. In contrast, a separate long-term study demonstrated a significant decrease in body weight when DWP16001 was administered continuously over 12 months. The observed trend toward insulin dose reduction suggests a potential insulin-sparing effect of DWP16001, which may contribute to reducing the risk of insulin-associated complications [16]. From a clinical perspective, a reduction in insulin requirements is advantageous, as it may lower the risk of insulin-induced hypoglycemia and enhance owner compliance with treatment protocols. These findings support the potential role of SGLT2 inhibitors as adjunctive agents in the management of canine diabetes mellitus. Fewer dogs in the DWP16001 group required an increase in insulin dose during the treatment phase of our study, and a slightly higher proportion experienced a dose reduction. Four dogs in the DWP16001 group developed asymptomatic hypoglycemia, which was resolved with insulin dose adjustments. Although the difference between groups was not statistically significant, this trend may reflect an increased risk of hypoglycemia when SGLT2 inhibitors are combined with insulin, as has also been reported in human medicine. Such risks emphasize the importance of individualized insulin adjustment and careful monitoring during the early phases of SGLT2 inhibitor use. In addition, while no cases of euglycemic diabetic ketoacidosis (euDKA) were observed in this study, the potential for euDKA—particularly in insulin-treated patients—has been described in human medicine and should be considered when using SGLT2 inhibitors clinically [19].

Physiological parameters, including body weight, BCS, and systolic blood pressure, remained stable for both groups throughout the study. No significant weight loss or clinical signs suggestive of volume depletion or hypotension were observed, and systolic blood pressure remained stable throughout the study, supporting the metabolic safety of DWP16001 during the 8 weeks of treatment. This contrasts with findings from some human studies that SGLT2 inhibitors are associated with modest weight reduction and mild volume depletion [20]. These effects are primarily attributed to caloric loss via glycosuria and fluid loss through osmotic diuresis. While generally well tolerated, such effects emphasize the importance of appropriate patient selection and careful monitoring, especially for older individuals or those at risk of dehydration. Stool consistency, which was assessed using the Canine Fecal Scoring System (7-point scale), also remained stable throughout the study. Most dogs in both groups maintained scores within the normal range (3–4), and no increase in soft stool (score ≥ 5) or diarrhea (score ≥ 6) was observed, indicating that DWP16001 did not result in gastrointestinal adverse effects related to fecal quality.

Urinary parameters were assessed to evaluate the renal effects of DWP16001. As expected, urine glucose concentrations were elevated due to the glycosuric action of the drug, yet there were no cases of diabetic ketoacidosis or clinically significant ketonuria, which was defined as persistent ketone positivity (≥ 2 + on urine dipstick) accompanied by hyperglycemia and/or clinical signs suggestive of ketosis or ketoacidosis. The UPC showed a decreasing trend in the treatment group, although the change was not statistically significant. This may suggest a mild renoprotective effect of DWP16001, which warrants further investigation in longer-term studies. Similar findings have been observed in human medicine, where SGLT2 inhibitors have provided consistent renal benefits, including reduction in albuminuria and slowing of estimated glomerular filtration rate decline in patients with type 2 diabetes. For instance, large-scale clinical trials using another SGLT-2 inhibitor such as canagliflozin and dapagliflozin have shown that SGLT2 inhibitors reduce the risk of kidney disease progression, independent of their glucose-lowering effects [21, 22]. These renoprotective outcomes are believed to result from improved glomerular hemodynamics, reduced intraglomerular pressure, and attenuation of inflammation and fibrosis. The mild decrease in UPC observed in this study, although not statistically significant, may reflect similar underlying mechanisms and highlights the potential utility of SGLT2 inhibitors in supporting renal function in diabetic dogs.

Hematological, serum biochemical, and blood gas parameters remained within reference ranges, and no significant adverse events were reported in either group. Clinical signs such as appetite, activity, and urination also remained stable, indicating that DWP16001 was well tolerated. In this study, a simplified clinical scoring system was employed to monitor changes in general health parameters such as appetite, activity, urination, and gastrointestinal signs. While this system has not been formally validated in diabetic dogs, it allowed for standardized assessment of potentially treatment-related effects and clinical stability throughout the study. We acknowledge that disease-specific scoring systems such as the ALIVE Diabetes Clinical Score (ALIVE DCS) provide a more comprehensive framework for evaluating clinical outcomes in canine diabetes and may be considered in future studies focusing on long-term disease management or comparative therapeutic efficacy [23].

Nevertheless, this study has several limitations that must be considered when interpreting the results. First, the observation period was relatively short at only 8 weeks. This limited timeframe restricts our ability to fully assess the long-term efficacy and safety of DWP16001, especially with respect to potential cumulative effects, sustained glycemic control, and renal or cardiovascular outcomes over a more extended period. Longer studies may reveal additional benefits or unanticipated side effects that were not apparent during this brief interval. Another limitation is the absence of an a priori sample size calculation or power analysis. As this study was exploratory in nature, the sample size was determined based on case availability and institutional feasibility. Future studies with larger sample sizes and appropriate power calculations are warranted to confirm and expand upon the findings reported here. In addition, the sample size in our study, although comparable to those of other veterinary clinical trials, may have been insufficient to detect more subtle differences in key outcome measures such as insulin dosage adjustments and secondary metabolic parameters. A larger cohort may provide greater statistical power to identify minimal but clinically relevant changes that may have implications for optimizing treatment protocols. Another limitation is that glycemic variability was not assessed in this study. While blood glucose curves were obtained and both fructosamine and HbA1c were measured to evaluate overall glycemic control, these markers do not capture short-term fluctuations in blood glucose levels. It is possible for values to fall within reference intervals despite clinically significant variability. These limitations suggest that further research with longer duration and increased sample size is necessary to validate and extend these results although the initial findings are promising.

In conclusion, DWP16001 is a promising adjunct therapy for diabetic dogs, especially those with poor glycemic control. It demonstrated a significant glucose-lowering effect without major adverse events and was generally well tolerated. The potential for insulin dose reduction and renal benefit, while encouraging, should be further evaluated in longer-term studies involving larger samples. The clinical use of DWP16001 should include careful glucose monitoring and individualized insulin adjustments to mitigate the risk of hypoglycemia.

## Data Availability

All data generated or analyzed during this study are included in this published article.

## Abbreviations

ACTH: Adrenocorticotropic hormone
ALP: Alkaline phosphatase
ALT: Alanine aminotransferase
ANOVA: Analysis of variance
AST: Aspartate aminotransferase
BCS: Body condition score
BUN: Blood urea nitrogen
CGM: Continuous glucose monitoring
GGT: Gamma-glutamyl transferase
HbA1c: Glycated hemoglobin
IRIS: International renal interest society
PO: Per Os (by mouth)
SD: Standard deviation
SDMA: Symmetric dimethylarginine
SGLT2: Sodium-glucose cotransporter 2
U: Unit
UPC: Urine protein-to-creatinine ratio
USG: Urine specific gravity
